# Implementation considerations for offering personal genomic risk information to the public: a qualitative study

**DOI:** 10.1186/s12889-020-09143-0

**Published:** 2020-06-29

**Authors:** Amelia K. Smit, Gillian Reyes-Marcelino, Louise Keogh, Kate Dunlop, Ainsley J. Newson, Anne E. Cust

**Affiliations:** 1 Faculty of Medicine and Health, Sydney School of Public Health, Cancer Epidemiology and Prevention Research, The University of Sydney, Sydney, Australia; 2 Faculty of Medicine and Health, Sydney School of Public Health, Sydney Health Ethics, The University of Sydney, Sydney, Australia; 3 Melanoma Institute Australia, The University of Sydney, Sydney, Australia; 4 Melbourne School of Population and Global Health, The University of Melbourne, Parkville, Australia

**Keywords:** Genomics, Population, Implementation, Prevention, Early detection, Risk

## Abstract

**Background:**

Genomic risk information, based on common genomic susceptibility variants associated with risk of complex diseases such as cancer, may be incorporated into personalised prevention and screening strategies. We aimed to engage with members of the public, who are important stakeholders in this process, to further inform program development and other implementation outcomes such as acceptability and appropriateness.

**Methods:**

Semi-structured interviews were undertaken with 30 participants (aged 24–69 years, 50% female) recruited from a pilot trial in which they received personalised genomic risk information for melanoma. We explored participants’ views and attitudes towards offering general personal genomic risk information to the broader population. The data were analysed thematically.

**Results:**

Two overarching themes relevant to implementation considerations were identified. Firstly, participants’ preferences for accepting an offer of genomic risk information were based on family history, disease incidence and the possibility of prevention. Secondly, participants felt that the processes for offering risk information should be based on individual preferences, triaged according to risk and be supported by a health professional trained in genomics.

**Conclusions:**

Participants felt that offering personal genomic risk information to the general population to inform prevention and early detection recommendations is acceptable, particularly for common, complex conditions such as cancer. Understanding participants’ preferences for receiving genomic risk information will assist with communication strategies and health workforce planning. We anticipate that these findings will contribute to the development of implementation strategies for incorporating genomic risk information into routine clinical practice.

## Background

Precision public health strategies aim to personalise prevention and screening recommendations by integrating individual risk assessments [1, 2]. Genomic risk information, based on common genomic susceptibility variants associated with the risk of complex diseases such as cancer, may be incorporated into individual risk assessment [3–6]. There is an important need to examine the efficacy, cost-effectiveness and ethical considerations prior to offering genomic risk information to the population [1, 7, 8]. Alongside this, evidence on implementation considerations will be essential to ensure effective and appropriate research translation [1, 9, 10].

The Consolidated Framework for Implementation Research (CFIR) enables the identification of determinants of implementation that are relevant to specific innovations or changes to healthcare systems and pathways [11, 12]. The CFIR has been adapted to create the Genomic Medicine Implementation Model that aims to evaluate the implementation of genomic medicine interventions in clinical practice [13]. In the context of population genomic testing for risk of cancer and other diseases, individuals’ expectations and preferences for receiving genomic risk information may impact the clinical and personal utility of integrating genomic risk information into prevention and early detection strategies [14]. Further, substantive engagement with relevant stakeholders such as the public can improve program development and other implementation outcomes such as acceptability and appropriateness [7, 15].

Several studies have examined the public’s views towards personal genomic testing via commercial direct-to-consumer providers [16, 17]. But there is limited research on the public’s attitudes, expectations and preferences for the provision of personal genomic risk information as part of population prevention and screening strategies, beyond breast cancer [18]. The aim of this research study was to address this research gap using qualitative interview methodology, one of the recommended approaches to measure CFIR constructs [13].

## Methods

### Recruitment

This is a sub-study of a pilot randomised control trial that examined the feasibility and acceptability of providing personalized genomic risk of melanoma to the public [19]. Participants in the trial were recruited via the Cancer Council New South Wales ‘Join a Research Study’ database. Participants (intervention and waitlist control) in the trial received personal genomic risk of melanoma information, a phone call from a genetic counsellor and educational information on melanoma prevention and early detection. On completion, 30 participants (of 41 invited) took part in a semi-structured interview. Participants were purposively sampled, ensuring different genders, ages and genomic risk (low, average, high) results were included. Ethics approval was obtained from The University of Sydney and participants gave informed consent. The conduct, design and reporting of this study follows the Standards for Reporting Qualitative Research [20].

### Data collection: semi-structured interviews

An interview guide was developed specifically for the broader qualitative substudy. This included a range of questions related to participants’ experiences in the pilot trial, and a subset of questions that explored their views on offering the type of melanoma risk information they received to the general population. This paper focuses on participant responses and discussion related to their attitudes, expectations and preferences towards offering genomic risk information for melanoma and other diseases to the broader population (see Additional file 1 for the subset of interview questions that are specific to these aspects). Results on the other data collected in the broader qualitative substudy (including the other subsets of questions in the interview guide from the broader study) have been published elsewhere [21, 22]. The interview guide was piloted with consumers and other researchers. The interviews were conducted by AKS, audio-recorded and transcribed.

### Data analysis: qualitative thematic analysis

We conducted thematic analysis, which involved developing a coding framework using a hybrid (deductive and inductive) approach; i.e. the transcript data were coded according to broad top-level categories that matched key topics in the interview guide, then we searched for patterns and ideas within the top-level categories and further coded the data into sub-categories and themes, which were developed inductively [23–25]. Throughout the coding process, the research team with diverse areas of expertise (implementation science, sociology, bioethics, and cancer epidemiology) discussed the coding which enabled critical reflection on the developing themes. Interviews continued until no new themes or ideas were identified in the data (i.e. data saturation was reached) [23]. Coding was undertaken by AKS, and GRM. Agreement between coders was high and discrepancies were discussed until consensus was reached. NVivo11 (QSR International) was used to facilitate the coding process.

## Results

### Participants

The characteristics of the 30 participants interviewed are show in in Table 1. The mean length of the broader interviews was 32 min (range 14–57 min), but only a portion of the broader interview time was specific to implementation considerations. Overarching themes specific to implementation considerations were identified in the thematic analysis, which related to preferences and rationale for offering genomic risk information and the mode of delivery.

**Table 1:** Qualitative interview participant characteristics

Characteristic	Interviews (*n* = 30)
Age, mean (range)	53 (24–69)
Female, N (%)	15 (50%)
Highest level of education	
High school or equivalent	7 (23%)
Trade/diploma	9 (30%)
University degree or higher	14 (47%)
Genomic risk category, N, (%)	
High	12 (40%)
Average	8 (27%)
Low	10 (33%)
Interview conducted via:	
Telephone	27 (90%)
Face-to-face	3 (10%)

### Themes

1. Preferences for receiving genomic risk information are based on family history, disease incidence and the possibility of prevention

Most participants reported that they would be interested in receiving personal genomic risk information, similar to the information they received in the pilot trial, for other conditions if it were available:

“*If there was a general program the public can be informed about their likelihood of having or becoming ill by one cause or another, yes*.” (Female, low genomic risk)

When prompted to reflect on why they would choose to receive genomic risk information for certain conditions, three sub-themes were identified:

1.1. Family history is a prompt

Family history of disease(s) was identified by many participants as a motivating factor to obtain personal genomic risk information because they were interested in understanding the genetic heritability of a disease, and how this related to their own risk.

*“I’m just sort of thinking about things where there is a strong family history (…) I guess mental illness particularly, depression, because that runs in the family as well. So I’d be interested to see what the genetic component is around that.”* (Female, average genomic risk)

1.2. Common conditions are preferred

Participants reported preferences for receiving genomic risk information for conditions (primarily cancers) that they perceived to be common in the population and relevant to their gender and age groups:

“[I would be interested in] *things like bowel cancer and (…) prostate cancer. I guess they’re the couple of the big ones aren’t they?”* (Male, average genomic risk)

1.3. Possibilities for disease prevention is a motivation

Another reason to obtain genomic risk information was the possibility of disease prevention through improved health-related behaviours:

*“I think that would be really good* [to receive this type of information]. *I guess if that other cancer is preventable, then you can try and change your behaviour or even if it’s not (…) there’s always something you can do, but I think just being aware of your risks, I guess you can look out for signs.”* (Female, low genomic risk)

2. Processes for providing the genomic risk information should be based on individual preferences and be perceived as trustworthy

Regarding how personal genomic risk information is presented to recipients, participants felt that individuals should be able to elect to receive it online or via postal mail as long as these options were secure:

“*I would think that in the future that the information should be available electronically to people, by email or by able to be logging into a secure website. But the option there, of course, for people who still would like to receive it through the old fashioned way* [via postal mail]*.*” (Male, low genomic risk)

Two subthemes were identified within this broader theme:

2.1. Triaged strategies for health professional involvement

Participants’ views on the level of health professional involvement in providing personal genomic risk information to the public varied. But most agreed that a health professional should be involved, at least as a point of contact:

*“I think people should, if they can have the option or if it comes through their general practitioner. Through some profession, I don’t think it should be some site that you just log in and you send off your DNA. I think it’s good to have that personal contact as well and advice from a professional*.” (Female, average genomic risk)

Many participants described triaged approaches for health professional contact according to individual risk:

*“Maybe you could have a two-tier approach to that, where people who are average or below the average risk, they get the opportunity to ring someone and perhaps people who come back as a higher or a high risk get a call.”* (Male, low genomic risk)

2.2. Health professionals should be equipped to provide genomic risk information Many participants felt that a general practitioner (GP) could provide straightforward genomic risk information. But if the information was more complex, a genetic counsellor should be involved.

“*I mean if GPs are just giving sort of just a general risk rating and some guidance on ways to change their behaviour, things to do, et cetera, then you probably don’t need a genetic counsellor involved; the GP can probably handle it*.” (Female, high genomic risk)

Some felt that GPs would be more accessible than genetic counsellors, in terms of physical access and costs to the individual:

*“I think if you want some more access, I guess, people are more likely to go to a GP than maybe - but then if it’s not something that’s for the public, it’s more like, I don’t know, genetic counselling, is that more of a private, more expensive option, so might not be as readily available.”* (Female, average genomic risk)

Participants did express some concerns about the limited time that is typically available in GP consultations and whether GPs had adequate knowledge and training in genetics:

“*I mean GPs are good at the general stuff of sun protection, but how good are they at going that little bit step further as far as DNA is concerned? I’m not sure about that*.” (Male, average genomic risk)

Many felt that other health professionals such as nurses or pharmacists could provide this type of information, as long as they were equipped to do so:

“*Well I think that you’d be giving it to people in the medical profession or a pharmacist or someone with experience in those areas with knowledge. See some nurses would be fantastic, you know, but as long as they’ve got a backup.*” (Male, high genomic risk).

## Discussion

This qualitative study explored a range of perspectives on the potential opportunities and challenges for the translation and incorporation of genomics-based disease risk information into population prevention and screening strategies. Our findings align with the Genomic Medicine Implementation Model, which emphasises the importance of understanding the knowledge and beliefs of otherwise healthy potential recipients of genomic risk information [13].

We found that participants’ rationales for receiving genomic risk information for themselves in the future were based on their family history of disease and the possibility of prevention or early detection for the conditions. Previous studies conducted among healthy individuals undertaking genome sequencing in research settings have found similar motivations [26, 27]. To date, most research studies on genomic interventions have focussed on diet changes, smoking cessation and physical activity, and have not found evidence of significant effect on behaviour change. But these studies were limited by high risk of bias and small sample sizes [28, 29]. Ongoing research is examining the behavioural and psychosocial impact of receiving personal genomic risk information for conditions such as melanoma [30]. Our qualitative findings in the current study highlight the importance of providing support and education on prevention and early detection alongside genomic risk information.

Participants in our study were interested in receiving personal risk information for conditions that they perceived to be relatively common, particularly cancer, although they were recruited via a cancer research database. Another Australian study conducted among cancer patients and their relatives found high enthusiasm for offering genome sequencing to the general population, prioritising those with a family history of cancer [31]. In a population sample not selected for their personal or family history of cancer, a Canadian study found stronger support for population genomic sequencing for colorectal cancer risk than type 1 diabetes, when conducted alongside existing screening strategies [14]. They also found that public evaluations of the acceptability of population genomic sequencing depended on the participants’ experiences of personal health decision making and the availability of interventions to reduce disease risk [14]. Other research has also shown that perceived benefits of including genomic information in population-based screening include the potential for early detection, prevention and closer monitoring [32]. It is important to note, however, that enthusiasm and optimism regarding hypothetical genomic testing may not necessarily translate into uptake of testing. This optimism may also be based on an idealistic view of the quality and benefits of genomic risk information, which is frequently represented in popular social discourses and mass media [33]. Factors such as ethnic minority status may also influence public views towards testing [34], although these were not explored in the present study. Future research could explore the factors that influence actual uptake of population-based genomic sequencing, including whether and how individuals’ views change after learning more about variability in data quality.

Participants in our study proposed triaged approaches to providing genomic risk information according to risk levels, which supports our previous findings [35]. Similarly, a cross-sectional study on United Kingdom women’s attitudes towards tailored breast cancer screening based on individual risk including genetic factors, found that letters or emails were preferred for very low-risk results and face-to-face communication was preferred for very high-risk results [36]. GPs were the most commonly preferred providers of face-to-face results [36]. In the present study, participants felt that a health professional should be involved in the information delivery process, even if only as a point of contact for those who seek further communication. This aligns with other research findings that suggest Australians are more comfortable seeking health-related genetic information from a health professional compared to a commercial company [37, 38]. However, some participants expressed a lack of trust in GPs providing genomic risk information, as reported by others for personalised breast cancer prevention information [39].

Participants also raised issues of access to genetic counsellors and costs for the individual. If genomic risk information is offered to the population, support or provision for those tested may be accessed through health professionals with genetic knowledge (for example nurses trained in genomics) and alternative modes should also be considered, such as genetic counselling chatbots [40]. In countries such as Australia, the genetic counselling workforce remains limited in size and may be best accessed when pre- and/or post-test counselling provision is required. Education of health professionals will be crucial to ensure an informed health professional workforce. Emerging research studies are examining additional strategies to overcome feasibility and access issues, such as telephone genetic counselling [41, 42]. A research study is examining various delivery models for the provision of predictive genetic testing to the public in Europe and the US, Canada, Australia and New Zealand [43]. The variability of health systems and payment models will also likely influence the provision of genomic risk information at the population level. This may depend on whether offering genomic risk information is incorporated into a formal population screening program (which could be publicly funded) or as a test offered in clinical practice (publicly or privately funded). Considerations such as these will impact workforce needs and training requirements, and require further implementation research.

There are some limitations to the generalisability of our findings. Participants in this study may have been particularly optimistic about offering personal genomic risk information to the general population because of their participation in a pilot trial in which they received their own personal risk. An advantage of this approach is that it provided participants with a lived experience of receiving genomic risk information, as opposed to relying only on hypothetical scenarios. The interview questions did not focus on specific diseases or existing screening programs, and public views may differ according to these factors or for diverse population subgroups. While these findings focus on the Genomic Medicine Implementation Model [13], there are other factors within and beyond this model that influence the provision of genomic risk information to the public, including referral visits, access, and costs.

## Conclusions

In conclusion, participants thought it was acceptable to provide personal genomic risk information to the general population to inform prevention and early detection recommendations, particularly for conditions such as cancer, and where prevention strategies can be implemented. They proposed tailoring the delivery of the personal risk information according to risk level, such that people at higher risk could receive their information via a genomics-trained health professional and those at lower risk receiving written correspondence (letter or email) with the option of contacting a health professional. These findings will contribute to implementation strategies for incorporating genomic risk information into population preventive healthcare.

## Supplementary information

**Additional file 1.** Interview guide questions specific to implementation considerations. This table contains the subset of questions from the interview guide specific to implementation considerations.

## Data Availability

The datasets used and/or analysed during the current study are available from the corresponding author, subject to approval from the ethics committee that approved the original study.

## Abbreviations

GP: General Practitioner
CFIR: Consolidated Framework for Implementation Research
