# Supplementary material for: Implementation considerations for offering personal genomic risk information to the public: a qualitative study
Source: BMC Public Health. 2020 Jun 29;20:1028. doi: 10.1186/s12889-020-09143-0 (PMC7325160; doi:10.1186/s12889-020-09143-0)
Supplement: Supplementary file 1 — Additional file 1. Interview guide questions specific to implementation considerations. This table contains the subset of questions from the interview guide specific to implementation considerations. [file 12889_2020_9143_MOESM1_ESM.docx]

**Additional file 1. Interview questions specific to implementation considerations.**

| **Questions and prompts** |
| --- |
| In this interview, we will talk about: your own thoughts and responses to your personalised melanoma genetic risk information, and your views about providing the public with this kind of information. There are no right or wrong answers to any of the questions that I ask today. If you’re not sure what I’m asking feel free to tell me and to ask any questions along the way.  Do you have any questions about this interview before we begin?   - Would you choose to receive genomic risk information for other conditions? Why/why not? - How do you think this type of information should be provided to the population? Why do you think that? - Should a health professional be involved in the provision process, and if so, what type of health professional? What makes you think that?   Closing comment: That’s everything I wanted to talk to you about today. Is there anything else you’d like to say or discuss?  Thank you for taking the time to talk to me on the phone/in-person today.  *Note: other questions were included in the interview guide but are reported on elsewhere* |
